# Supplementary figures and images for: Prioritizing and characterizing functionally relevant genes across human tissues
Source: PLoS Comput Biol. 2021 Jul 16;17(7):e1009194. doi: 10.1371/journal.pcbi.1009194 (PMC8284802; doi:10.1371/journal.pcbi.1009194)

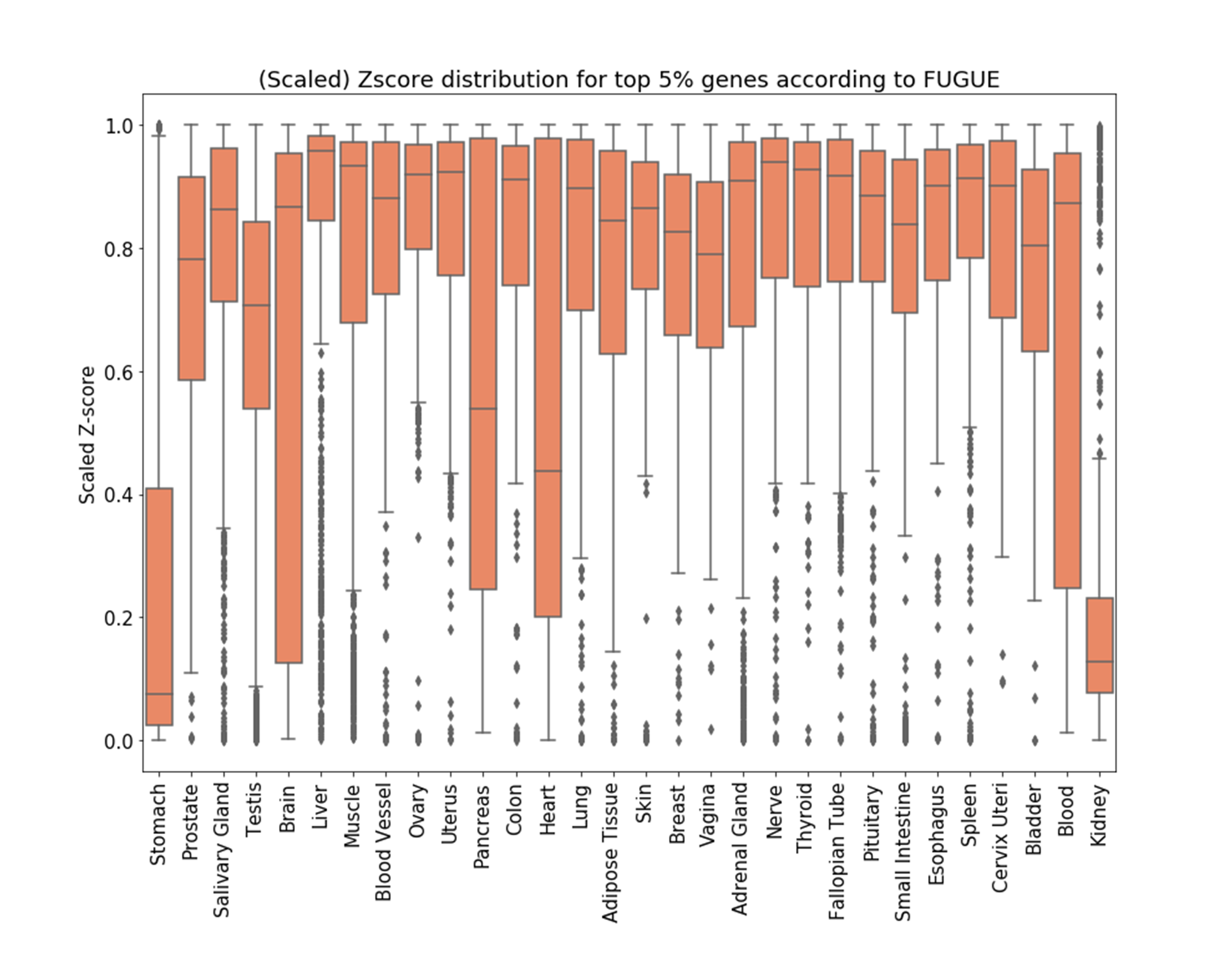

Supplement: S2 Fig — (TIF) [file pcbi.1009194.s002.tif]
