# Supplementary material for: Prioritizing and characterizing functionally relevant genes across human tissues
Source: PLoS Comput Biol. 2021 Jul 16;17(7):e1009194. doi: 10.1371/journal.pcbi.1009194 (PMC8284802; doi:10.1371/journal.pcbi.1009194)
Supplement: Attachment — Submitted filename: Somepalli-PloSCB-Response.docx [file pcbi.1009194.s012.docx]

Dear Zhaolei and Jian,

Thank you very much for overseeing the review of our manuscript. We are grateful to the reviewers for their kind and thoughtful comments. We are very encouraged to see that the reviewers are positive across the board. The comments are by and large minor, which we have now addressed and updated our manuscript accordingly. We have included the point-by-point response below. We hope that you and the reviewers find the revised manuscript suitable for publication in PLoS Comp Bio.

Best regards

-Sridhar

Reviewer #1: In this paper, the authors intend to predict the genes that are likely relevant to a tissue’s function, which is defined as inactivation of the gene has a phenotypic effect observable at the tissue level. The authors tested 14 features and then used them in a machine learning model called FUGUE to predict tissue relevant genes. The authors validated the predictions by examining the genes’ functions and location on the genome.

The work seems to be significant because most current research focus on either cellular level or organism level, while research on the relevance on the tissue level is lacking. However, there are major concerns that need to be addressed before the paper can be accepted.

1. The authors’ proposal to use genes that don’t express in certain tissues as the negative training set seems problematic. Can the authors provide information on how many genes are expressed and not expressed in each type of tissues? The most meaningful predictions should be to differentiate relevant genes from all genes that are expressed in a tissue. Using genes that don’t express in a tissue might cause the algorithm to recognize features that are not truly responsible for the “relevance” of the genes.

This is a very good point and indeed something we thought about when designing our study. On the one hand, as the reviewer says, we would like to develop a model that can discriminate functional genes from non-functional ones based on features beyond the expression. On the other hand, we reasoned that, our ultimate goal is to prioritize genes in a sample using any and all information - hence our choice.

We would like to clarify that we construct our negative (N) set based on the lack of detectable proteins in the HPA database and not based on mRNA expression. Interestingly, the genes in N exhibit a range of mRNA expression values in the GTEx dataset, and notably, in some tissues the mean expression of genes in N are comparable or even greater than the mean expression in the positive (P) set. As suggested, we have now added Supplementary Table S1 (mentioned at line 122) showing the sizes and mean expressions of N, P, and U sets in all tissues. We have now clarified this in line 156.

We had shown in the manuscript (Fig 2D) that the model based on Network-only features performs as well as the model based on expression-only features and a combined model outperforms the expression-only model, suggesting that the model is learning features beyond expression.

In the manuscript, we had also mentioned (line 159) that the model built on the Z-score-matched subsampled data still has a reasonably high AUC score of 0.8.

As per the reviewer’s suggestion, we have now added auROC and auPRC results for P vs U (representing the genes that have detectable proteins in the tissue in HPA), showing a slight drop in performance relative to P~N, as expected, but nevertheless reasonably high auROC of 0.78 and auPRC  observed/expected auPRC = 4.6. We have now added this additional analysis at line 163.

2. It is a little surprising to see among all 14 features, 12 achieves statistical significance to differentiate positive and negative sets. Can authors provide biological meanings on why these features work? Most of the features are related to gene expressions, but in negative training sets, the gene expression is essentially 0 since they don’t express in the tissues. Could this be the reason why features based on gene expression all work well?

Thanks for asking this. Indeed, unlike many machine learning approaches that are based on large agnostic collection of features, all the features we used are based on specific biological rationale and are a priori expected to discriminate the functional genes. We have mentioned these rationales in the Methods section. Half of the features in fact include network information and when they do rely on the expression, they rely on the expression of the neighboring genes and not the gene in question. As mentioned in response to the comment above, we had shown in the manuscript (Fig 2D) that the model based on Network-only features performs as well as the one based on expression-only features and a combined model outperforms the expression-only model, suggesting that it is learning features beyond expression of the gene of interest. The response to the comment above addresses this issue more comprehensively, and we have made several edits to the manuscript accordingly as mentioned above.

3. I don’t see the authors list the number of TRGs predicted for each issue. Perhaps the authors could provide this information?

Our model provides a priority score for every gene in a given sample/tissue. While we have done most of our downstream analysis based on the top 5% or 10% of the genes sorted by the priority score for expedience, we had in fact provided the priority score for ALL genes in each tissue in Data File 1, as mentioned in the manuscript at line 200. We thought it more useful to provide the entire priority list rather than recommending an arbitrarily chosen threshold to define TRGs. We have now further clarified this point at line 200.

Reviewer #2: Somepalli et al developed a machine learning model – FUGUEO, aiming to predict tissue-relevant genes. They combined 14 features in a XGBoost (gradient boosted decision tree) model using data either from tissue-specific gene expression or tissue-specific protein interaction networks. They demonstrated that FUGUE can discriminate between evidence-based known positive and negative gene-tissue pairs, with an average auROC score of 0.86 and auPRC of 0.50, based on 5-fold cross validation. Moreover, they evaluated the performance by analyzed independent datasets from 1) a list of known genes relent to phenotypic aberration in relevant tissues and 2) gene expression data generated from 317 human Embryonic Stem Cell (hESC) transcriptome with additional information of the 500 essential genes from knock-out experiments in hESC. Overall, they provided additional evidence to support the tissue-relevant genes prioritized based on FUGUE. For the downstream analyses, they additionally showed that the identified tissue-relevant genes may be relent to tissue development, structure, and cancer oncogenesis.

Overall, I think this is a well-conducted study with reasonable motivation to address the challenge of identifying tissue-relevant genes. The developed FUGUE tool could be useful in the related filed. However, I have specific comments/concerns below:

1) When they applied FUGUE to the data from hPSC, They found that a conventional approach Z-score ranks of the essential genes was higher than the FUGUE ranks. This could be a major limitation in their approach. It’s still unclear whether FUGUE may outperform previous approaches in various conditions, while further discussion are needed.

The reviewer is correct and indeed we had discussed this in the manuscript.

Note that we had performed an independent testing for each tissue where data from one tissue was entirely excluded from training. This mimics the scenario when FUGUE is applied to a novel context. We had earlier reported FUGUE performance for each tissue. Now, in addition, we directly compared FUGUE and Z-score ranks for the positive genes in each tissue (excluded from the training set). Of the 25 tissues with positive genes, we found that in 11 tissues FUGUE ranks were better (P  < 0.05) than Z-score ranks. Notably, Z-score ranks were not better than FUGUE ranks in any of the tissues; the numbers are 9 out of 13 tissues if we only consider tissues with at least 100 positive genes. This shows that FUGUE often performs better than the conventional approach in independent datasets. We have now included this new result at line 177.

As far as our hPSC application is concerned, it is entirely possible that the rules governing the gene functionality are different in ESC/hPSC than they are in differentiated tissues. Indeed, as we had noted, when we cross-validated the model within hPSC (instead of imposing the model trained on differentiated tissues), FUGUE does outperform the conventional approach. We have now performed one additional test.

In the scenario where we train FUGUE on GTEx and test on hPSC, we have now assessed whether superior performance of Z-score might be because of a small fraction of genes that are highly specific to hPSC (i.e., highest Z-scoring genes). We found that if we exclude the genes with top 5% Z-scores, then the FUGUE ranking is higher than Z-score rankings (P = 2E-4) for the remaining genes, and if we exclude top 10% genes with highest Z-score then the signal gets even stronger (1E-10). This suggests that FUGUE may have value even in independent datasets beyond the most specifically expressed genes. We have added this new finding at line 236.

We have now further clarified this issue while highlighting the potential limitation at line 406.

2) The authors analyzed the data from Topologically Associated Domains (TADs) and showed additional evidence that the identified tissue-relevant genes were enriched in TADs. However, the findings did not really provide a direct evidence to support their hypothesis. They should analyze tissue-specific enhancers which have been released the Roadmap project or the most recent literature from the EpiMap project.

Thank you for this suggestion. A lack of tissue-specific TADs and also a general lack of tissue-specificity in TADs, as previously reported, had precluded us from doing this analysis. In addition, the TAD analysis is partially redundant with our results showing that TRGs tend to be clustered on the genome. As suggested by the reviewer, we have now leveraged the recently reported tissue-specific enhancer-gene maps in EpiMap. We obtained the tissue-specific gene-enhancer maps for the 8 tissues that could be unambiguously mapped between EpiMap and our study - Brain, Kidney, Heart, Liver, Lung, Muscle, Pancreas, and Spleen. We expect TRGs to be under greater tissue-specific transcriptional control, i.e., interacting with a greater number of enhancers. We tested this by comparing the number of enhancers linked with TRGs with random gene sets. In 7 of the 8 tissues (except Brain), the tests strongly support our expectation. We have now reported this at line 374.

3) They mapped to genes from GWAS-identified loci. It’s unclear how to link target genes for GWAS-identified variants (i.e. eQTL analysis in specific tissues or just location-based analysis). Should clarify this.

We regret the lack of clarity. While there are multiple approaches used in the literature to estimate gene-centric association scores, we followed a stringent approach where only the SNPs within the gene locus were used to associate a gene to a trait. We have now clarified this in the Methods section at line 544.

4) In Figure 4, it will be helpful to provide the information tissue-relevant transcription factors to facilitate the review.

Thank you for this suggestion. We have now updated Fig. 4 to include tissue-relevant transcription factors and updated the figure legend.

Reviewer #3: In this manuscript the authors applied a machine learning method FUGUE to identify the genes that are highly tissue-specific. The model applies features from transcriptomic data and protein interaction network. They achieve quite a remarkable cross-validated auROC score and the results are replicated in independent datasets. They also provide ranked lists of these tissue relevant genes (TRGs) in 30 human tissues as well as they offer an open-source software application.

In my opinion this is a well written manuscript, clearly depicting the goals of the study and walking the reader throughout the entire journey from the limitations of the previous studies, to their novel approach and the data limitations that impact the interpretations of their results. The methods applied are rigorous and fully support the conclusions.

Fig 1. Perhaps some revisiting of this figure to make it more readable. It took me a bit to read the caption and follow the arrows to understand the process. I’d suggest perhaps to give more of a top-bottom or left-right flow.

We have now updated Fig. 1 and the legend to make it easier to follow.

Fig 2A: what tissue has the average positive score very close to the negative one?

Thanks for pointing this out. It is the Pituitary gland, and as it happens this tissue has the fewest number (2) of Negative genes, and only 65 Positive genes. It is clearly an outlier. We have indicated this in line 174.

KRAS is an important gene in many cancers, but often in colorectal adenocarcinoma. Was there any association in addition to ovarian?

Thank you for pointing this out. We checked the FUGUE ranking of KRAS across tissues, and indeed, KRAS normalized rank was above 0.9 in 13 tissues, including colon where it was 0.97. We have now mentioned this at line 224.

Data_File_2: “Table S1” is labeled as Table S2 in the gray box.

We have now fixed this.
